# Elevated central venous pressure is associated with increased mortality in pediatric septic shock patients

**DOI:** 10.1186/s12887-018-1059-1

**Published:** 2018-02-13

**Authors:** Seung Jun Choi, Eun-Ju Ha, Won Kyoung Jhang, Seong Jong Park

**Affiliations:** Division of Pediatric Critical Care Medicine, Department of Pediatrics, Asan Medical Center Children’s Hospital, University of Ulsan College of Medicine, 388-1 Pungnap-2 dong, Songpa-gu, Seoul 138-736 Republic of Korea

**Keywords:** Central venous pressure, Mortality, Pediatrics, Septic shock

## Abstract

**Background:**

Central venous pressure (CVP) is an important factor affecting capillary blood flow, and it is associated with poor outcomes in adult septic shock patients. However, whether a similar association exists in pediatric patients remains unclear.

**Methods:**

We retrospectively analyzed data from patients admitted to our pediatric intensive care unit (PICU) between February 2009 and July 2015. Patients were divided into two groups—survivors and nonsurvivors—according to 28-day mortality. The associations between (a) mortality and CVP at 6, 24, 48, and 72 h after initiating treatment for established septic shock was analyzed and (b) initial serum lactic acid levels and 6-h CVP.

**Results:**

Two hundred twenty-six patients were included in this study, and the mortality rate was 29.6% (67 deaths, nonsurvivor group). Initial serum lactic acid levels, Pediatric Risk of Mortality (PRISM) III score, and Vasoactive–Inotropic Score (VIS) within 24 h after PICU admission were significantly higher in the nonsurvivors than in survivors (1.3 [0.9, 2.4] vs. 3.9 [1.6, 8.0] mmol/l, 11.0 [7.0, 15.0] vs. 17.0 [10.0, 21.5], 12.0 [7.0, 25.0] vs. 22.5 [8.0, 55.0], respectively with *p*-values < 0.001, < 0.001, and 0.009, respectively). In addition, compared to survivors, a greater percentage of nonsurvivors required mechanical ventilation (92.5% vs. 51.6%, *p* <  0.001) and showed a greater extent of fluid overload at 48 h after admission (3.9% vs. 1.9%, *p* = 0.006), along with higher 6-h CVP (10.0 [7.0, 16.0] vs. 8.0 [5.0, 11.0] mmHg, *p* <  0.001). Patient survival according to levels of CVP (CVP < 8 mmHg, CVP 8–12 mmHg, or CVP > 12 mmHg) showed that the CVP > 12-mmHg group had significantly greater mortality rates (50.0%, *p* = 0.002) than the other groups (21.3% and 27.5%). Furthermore, multivariate analysis identified significant associations of CVP > 12 mmHg, serum lactic acid levels, and the need for mechanical ventilation with mortality (OR: 2.74, 1.30, and 12.51, respectively; 95% CI: 1.11–6.72, 1.12–1.50, and 4.12–37.96, respectively).

**Conclusions:**

Elevated CVP is an independent risk factor for mortality in pediatric septic shock patients.

## Background

Central venous pressure (CVP) is frequently used to monitor the complex circulatory status of critically ill patients [1]. Although the utility of CVP is currently being questioned, [2, 3] it is still widely used as an indicator of hemodynamic status, and achieving a CVP > 8 mmHg is considered standard policy during fluid resuscitation (Early Goal Directed Therapy, EGDT) [4].

CVP is affected by cardiac function and blood volume returning to the heart [5, 6], and CVP elevation, which is frequently observed in critically ill patients, is associated with disturbances in venous return and microcirculatory blood flow [7]. Reports in recent literature suggest that alterations in microcirculatory blood flow are associated with poor outcomes in adult patients with severe sepsis [8–10], and an association between elevated CVP and mortality in adult septic shock patients has been studied [11–13]. However, no studies have examined the association between CVP and clinical outcomes in pediatric septic shock patients. Thus, we aimed to elucidate the association between CVP and mortality in pediatric septic shock patients admitted to a pediatric intensive care unit (PICU). In addition, we sought to analyze the correlation between CVP and serum lactic acid levels, which is another important parameter that reflects microcirculatory blood flow.

## Methods

A retrospective medical review of patients admitted to the 14-bed, level-III PICU at the Asan Medical Center Children’s Hospital, Seoul, Korea from February 2009 to July 2015, was performed. Septic shock was defined as sepsis accompanied by cardiovascular dysfunction [14], and only patients definitively diagnosed with septic shock were included in the study. The mainstay of treatment for septic shock at our unit is based on the Surviving Sepsis Campaign (2008, updated in 2012) [4, 15].

The patients were divided into two groups (survivors vs. nonsurvivors) according to 28-day mortality. Demographic information, data on laboratory results, clinical status, including comorbidities and sources of infection, the severity of illness based on the Pediatric Risk of Mortality III (PRISM III) score, Vasoactive–Inotropic Score (VIS), duration of PICU stay, and the requirement for mechanical ventilation. The VIS was calculated as follows [16]:

VIS = Dopamine dose (mcg/kg/min) + Dobutamine dose (mcg/kg/min) + 100 × Epinephrine dose (mcg/kg/min) + 10 × Milrinone dose (mcg/kg/min) + 10,000 × Vasopressin dose (units/kg/min) + 100 × Norepinephrine dose (mcg/kg/min).

The CVP at 6, 24, 48, and 72 h after identification and initiation of treatment for septic shock (henceforth abbreviated as 6-h CVP, 24-h CVP, 48-h CVP, and 72-h CVP, respectively) and the extent of fluid overload during the 3 days following initiation of treatment for septic shock were also ascertained. The following equation was adopted for calculating the extent of fluid overload (%):

{[Total fluid intake (L) – total fluid output (L)]/ICU admission body weight (kg)} * 100 (%).

According to the Surviving Sepsis Guidelines (2), study participants were divided into three groups: (1) CVP < 8 mmHg; (2) CVP 8–12 mmHg; and (3) CVP > 12 mmHg at each time point of CVP measurement, i.e., 6, 24, 48, and 72 h after admission. We then analyzed whether the patients with CVP in the recommended range (8–12 mmHg) had a survival advantage over the other groups.

SPSS software (ver. 18) was used for statistical analysis. Continuous and categorical variables were compared between survivors and nonsurvivors, and analyzed using the Wilcoxon rank sum test or the chi-square test, as applicable. The one-way analysis of variance and Bonferroni post-hoc test were used to compare the three CVP-based groups and the odds ratios in the CVP > 12-mmHg group. The Kruskal–Wallis test was used to compare serum lactic acid levels in the three CVP groups. Variables selected according to their *p*-values in the univariate analysis were tested in a multivariate logistic regression model. A p-value < 0.05 was considered significant for all analyses.

This study was approved by the institutional review board of the Asan Medical Center. The need for informed consent was exempted because of the retrospective nature of the study.

## Results

Our records indicated that 278 patients with a diagnosis of septic shock had been admitted to the PICU during the study period. Among these, patients without CVP measurements or those without CVP data within 6 h of diagnosis (*N* = 19), patients who died within 72 h after admission to the PICU (*N* = 14), patients who did not fulfill the above definition of septic shock (*N* = 11), and patients with incomplete CVP records (*N* = 8) were excluded. Therefore, data from 226 patients were used in this study. Catheters were predominantly inserted in the femoral vein (*n* = 188, 83.2%), followed by the internal jugular (*n* = 21, 9.3%) and the subclavian (*n* = 17, 7.5%). Of the 226 patients included, 67 died, resulting in a mortality rate of 29.6% and these patients formed the nonsurvivor group. The remaining 159 patients formed the survivor group.

Compared to the survivors, the median [IQR] PRISM III score and the VIS within 24 h of admission were significantly higher in the nonsurvivors (11.0 [7.0, 15.0] vs. 17.0 [10.0, 21.5] and 12.0 [7.0, 25.0] vs. 22.5 [8.0, 55.0]; *p* <  0.001 and *p* = 0.009, respectively), and a greater portion of the nonsurvivors required mechanical ventilation (92.5% vs. 51.6%, p <  0.001). Moreover, the median [IQR] lactic acid levels obtained immediately after admission was higher in the nonsurvivors than in the survivors (3.9 [1.6, 8.0] mmol/L vs. 1.3 [0.9, 2.4] mmol/L, *p* <  0.001). The extent of fluid overload within the first 24 h did not vary significantly among the groups, but was significantly greater in nonsurvivors than in survivors at 48 h (3.9% vs. 1.9%, *p* = 0.006). The 6-h CVP was significantly lower in survivors than in nonsurvivors (8.0 [5.0, 11.0] vs. 10.0 [7.0, 16.0)] mmHg, p <  0.001; Table 1).

**Table 1:** Patient characteristics

Characteristics	Survivors (*n* = 159)	Non-survivors (*n* = 67)	*p* value
Age, years	4.0 (0.6, 12.7)	4.6 (0.8, 14.4)	0.273
Male, n (%)	83 (52.2)	33(49.3)	0.771
Coexisting conditions, n (%)			
Cardiac disease	44 (27.7)	12 (17.9)	0.132
Hemato-oncologic disease	32 (20.1)	19 (28.4)	0.222
Neurologic disease	25 (15.7)	7 (10.4)	0.404
Pulmonary disease	20 (12.6)	13 (19.4)	0.216
Chronic renal disease	20 (12.6)	7 (10.4)	0.667
Gastrointestinal disease	12 (7.5)	6 (9.0)	0.789
Endocrinologic disease	6 (3.8)	3 (4.5)	0.805
PRISM III score	11.0 (7.0, 15.0)	17.0 (10.0, 21.5)	< 0.001
Duration of PICU stay (days)	12.0 (7.0, 35.0)	11.5 (4.0, 18.0)	0.062
Mechanical ventilation, n (%)	82 (51.6)	62 (92.5)	< 0.001
VIS	12.0 (7.0, 25.0)	22.5 (8.0, 55.0)	0.009
Lactic acid at admission, (mmol/l)	1.3 (0.9, 2.4)	3.9 (1.6, 8.0)	< 0.001
Brain natriuretic peptide (pg/mL)	286.0 (62.5, 1015.0)	461.0 (74.0, 1624.5)	0.271
Proven microorganisms, n (%)	91 (57.2)	45 (67.2)	0.183
Gram-positive	52 (32.7)	26 (38.8)	0.444
Staphylococci	24 (15.1)	14 (20.9)	0.331
Streptococci	10 (6.3)	4 (6.0)	0.928
Enterococcus	15 (9.4)	7 (10.4)	0.814
Other	3 (1.9)	1 (1.5)	0.837
Gram-negative	34 (21.4)	13 (19.4)	0.858
Klebsiella	15 (9.4)	2 (3.0)	0.106
Pseudomonas	5 (3.1)	3 (4.5)	0.697
E.coli	5 (3.1)	4 (6.0)	0.456
Acinetobacter	6 (3.8)	4 (6.0)	0.488
Other	3 (1.9)	0 (0)	0.557
Fungi	5 (3.1)	6 (9.0)	0.088
Candida	5 (3.1)	6 (9.0)	0.088
CVP at 6 h, mmHg	8.0 (5.0, 11.0)	10.0 (7.0, 16.0)	< 0.001
Fluid overload, %			
0–24 h	0.7 (−0.6, 2.6)	1.3 (−0.2, 3.6)	0.100
0–48 h	1.9 (−0.5, 5.7)	3.9 (0.9, 9.3)	0.006
0–72 h	3.8 (0.3, 7.9)	5.1 (1.8, 10.9)	0.055

The results are presented as the median (interquartile range) or as the number (%). *PICU*, Pediatric Intensive Care Unit; *PRISM III*, Pediatric Risk of Mortality III; *VIS*, Vasoactive Inotropic Scores

Patients were divided into three groups according to 6-h CVP and survival compared among them (Table 2). Mortality in the CVP > 12-mmHg group (50.0%) was significantly higher than that in the CVP < 8 mmHg (21.3%) or CVP 8–12 mmHg (27.5%) groups, but mortality was not significantly different between the CVP < 8-mmHg group and the CVP 8–12-mmHg group. Next, patients were consecutively divided into three CVP groups for each time point (6, 24, 48, and 72 h) as described above (Table 3) and the adjusted odds ratio for the group with CVP > 12 mmHg for each time point (h) was obtained. The odds ratio was low in CVP < 8 mmHg and CVP 8–12-mmHg groups than in the CVP > 12-mmHg group.

**Table 2:** Survival according to the 6 h CVP

	CVP < 8 mmHg^a^	CVP 8–12 mmHg^b^	CVP > 12 mmHg^c^
Total	89 (100.0)	91 (100.0)	46 (100.0)
Survivors	70 (78.7)	66 (72.5)	23 (50.0)
Non-survivors*	19 (21.3)	25 (27.5)	23 (50.0)

*p < 0.05 between ^a^ and ^c^, *p* < 0.05 between ^b^ and ^c^, but *p* > 0.05 between ^a^ and ^b^

The results are presented as the number (%)

**Table 3:** Odds ratio for death based on CVP values

CVP group	Adjusted Odds Ratio versus CVP > 12 mmHg
6 h	
CVP < 8 mmHg	0.452 (0.288–0.707)
CVP 8–12 mmHg	0.539 (0.341–0.854)
24 h	
CVP < 8 mmHg	0.298 (0.184–0.485)
CVP 8–12 mmHg	0.481 (0.292–0.791)
48 h	
CVP < 8 mmHg	0.299 (0.178–0.502)
CVP 8–12 mmHg	0.420 (0.247–0.712)
72 h	
CVP < 8 mmHg	0.371 (0.221–0.621)
CVP 8–12 mmHg	0.512 (0.294–0.892)

*CVP*, central venous pressure Odds ratios are shown with associated 95% confidence intervals

**Fig. 1:**
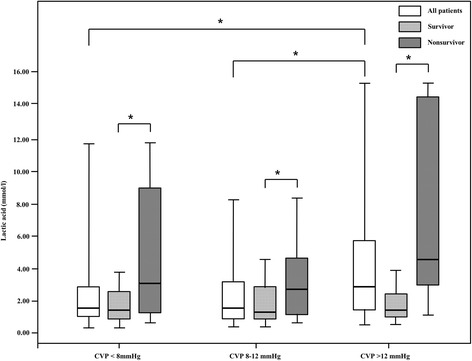
Boxplots of initial lactic acid levels at admission compared among three groups (CVP < 8 mmHg, 8–12 mmHg, and > 12 mmHg) *For differences in lactic acid levels in each of the CVP groups; *p* <  0.05 was considered to be statistically significant

Next, lactic acid levels at admission was compared among the three 6-h–CVP groups (Fig. 1), and was not significantly different between the CVP < 8 mmHg and CVP 8–12-mmHg groups (1.5 [1.0, 2.7] mmol/L vs. 1.5 [0.8, 3.2] mmol/L, *p* = 0.952). However, the CVP > 12-mmHg group had significantly higher lactic acid levels than in the other groups (2.7 [1.4, 5.9] mmol/L, *p* = 0.001 and *p* = 0.002, respectively). Furthermore, lactic acid levels was greater in nonsurvivors than in survivors, when analyzed within each of the three 6-h–CVP groups (1.4 [0.9, 2.3] vs. 2.9 [1.2, 8.8], *p* = 0.009 in the CVP < 8-mmHg group; 1.3 [0.9, 2.6] vs. 2.4 [1.1, 4.6], *p* = 0.021 in the CVP 8–12-mmHg group; and 1.4 [1.0, 2.1] vs. 5.3 [2.8, 14.4], *p* <  0.001 in the CVP > 12-mmHg group). Variables with significant association in univariate analyses were analyzed in a multivariate logistic regression model (Table 4), which revealed that CVP > 12 mmHg, lactic acid levels, and requirement for mechanical ventilation were the factors significantly associated with mortality with odds ratios (95% CI) of 2.74 (1.11–6.72), 1.30 (1.12–1.50), and 12.51 (4.12–37.96), respectively.

**Table 4:** Multivariate analysis of the independent risk for mortality

Variables	Adjusted odds ratio	95% confidence interval	*p* value
CVP > 12 mmHg	2.74	1.11–6.72	0.028
0–48 h fluid overload	0.98	0.92–1.05	0.709
VIS	1.00	0.98–1.01	0.974
Lactic acid	1.30	1.12–1.50	< 0.001
PRISM	1.05	0.99–1.11	0.057
Mechanical ventilation	12.51	4.12–37.96	< 0.001

*CVP*, central venous pressure; *PRISM III*, Pediatric Risk of Mortality III; *VIS*, Vasoactive Inotropic Scores

## Discussion

From a retrospective analysis of 226 cases, we showed that elevated CVP is associated with increased mortality in pediatric septic shock patients. In particular, mortality risk increased by > 2-fold in patients with CVP > 12 mmHg compared to those with minimally elevated CVP. Consistent with previous studies in the pediatric population [17–23], we also found that univariate analyses identified the extent of fluid overload at 48 h after admission, initial lactic acid levels, PRISM III score, VIS, and the requirement for mechanical ventilation as factors associated with mortality. However, only CVP > 12 mmHg, lactic acid, and requirement for mechanical ventilation remained significant in multivariate analysis.

In our study, the 6-h CVP was lower in the survivors, which is consistent with the results from previous studies in adult patients that showed that mean 6-h and 48-h CVP was lower in survivors than in nonsurvivors [12]. Furthermore, at all time points tested (6 h, 24 h, 48 h, and 72 h),the association between CVP and mortality showed that the CVP < 8-mmHg group had a higher survival rate than the CVP > 12-mmHg group. This is also consistent with previous results that show that patients whose 12-h CVP was < 8 mmHg had the highest survival rate, whereas those patients whose 12-h CVP was > 12 mmHg had the lowest survival rate [11].

There was no significant difference in mortality rate between the groups CVP < 8 mmHg and CVP 8-12 mmHg. Although EGDT is still considered the standard of care in managing severe sepsis and septic shock patients [4], recent studies have failed to yield similar positive results [24–27]. Septic shock is the resultant of not only volume depletion but also profound vasodilation along with microcirculatory dysfunction [28]. Moreover, some studies have questioned the usefulness of CVP in evaluating response to fluid resuscitation [29]. Therefore, although it may not be necessary to establish a specific target for CVP to guide fluid resuscitation, it may be more appropriate to interpret CVP elevation (particularly if the CVP was > 12 mmHg, as in our study) as a warning sign of impaired microcirculation.

In addition, we also analyzed the association between the elevated CVP and serum lactic acid levels, an indicator of the patients’ microcirculatory status. According to Vellinga et al., the percentage of small, perfused vessels was significantly lower in the high-CVP group (> 12 mmHg) than the low-CVP group (≤12 mmHg), which reflects the cardinal role of CVP in regulating microcirculatory perfusion during sepsis [30]. That organ perfusion and function (e.g., kidney, liver, and lung) are determined by CVP was also underscored by Wang et al. [13]. Although we found that there was no significant difference in lactic acid levels between the CVP < 8-mmHg and CVP 8–12-mmHg groups, the CVP > 12-mmHg group showed a higher lactic acid levels, which may be attributable to impaired microcirculatory blood flow and decreased organ perfusion in these patients.

Fluid overload has also been mentioned as a factor associated with poor outcomes in previous studies [31–35], and is in line with our results from univariate analyses. Although the statistical significance of fluid overload was not retained in multivariable analysis, it is undeniable that overall volume status is an important factor in assessing patients and making appropriate treatment decisions in clinical settings. Therefore, we think it wise to assess the intravascular and microcirculatory status, and simultaneously but cautiously interpret CVP numbers, while keeping in mind that multiple factors other than volume status also affect CVP.

Our study is limited by its retrospective nature and small sample size, as the data pertain to a single PICU. The retrospective nature of this study also prevented us from obtaining information on heart function, intraabdominal pressure, or intrathoracic pressure, all of which can also affect CVP. Therefore, we were not able to investigate the etiology of elevated CVP. However, we found that elevated CVP was indeed the parameter associated with poor outcome, which may serve as a valuable pivotal point in septic shock treatment in the pediatric population. Third, we did not analyze the effects, if any, of the different types of intravenous fluids used for volume resuscitation, as the impact of fluid volume and type in septic shock patients on mortality has been previously addressed [36]. However, as there is no consensus regarding the most appropriate fluid type for resuscitation other than crystalloid, we deem this an issue to be pursued in future research. Finally, future studies are required to validate our results in addressing the association between CVP and mortality on a larger scale using randomized, prospective studies.

## Conclusions

We showed that an elevated CVP (particularly if CVP > 12 mmHg) was associated with increased mortality in pediatric septic shock patients, and that further studies are required to analyze the difference in the outcome, if any, between groups with CVP < 8 mmHg and 8–12 mmHg.

## Abbreviations

CVP: Central venous pressure
EGDT: Early Goal Directed Therapy
ICU: Intensive care unit
PICU: pediatric intensive care
PRISM: Pediatric Risk of Mortality
VIS: Vasoactive–Inotropic Score
